# Trauma-induced acquired reactive perforating collagenosis^☆^^☆☆^

**DOI:** 10.1016/j.abd.2020.06.022

**Published:** 2021-03-20

**Authors:** João Renato Vianna Gontijo, Florentino Fernandes Júnior, Luciana Baptista Pereira, Moisés Salgado Pedrosa

**Affiliations:** a Dermatology Service, Hospital das Clínicas, Universidade Federal de Minas Gerais, Belo Horizonte, MG, Brazil; b Dermatology Service, Hospital Mater Dei, Belo Horizonte, MG, Brazil; c Private clinic, Belo Horizonte, MG, Brazil; d Faculty of Medicine, Universidade Federal de Minas, Belo Horizonte, MG, Brazil; e Patology Service, Hospital das Clínicas, Universidade Federal de Minas Gerais, Belo Horizonte, MG, Brazil

Dear Editor,

A 58-year-old female patient was referred for evaluation of lesions on the lower limbs that had been present for 30 days and had started following a minor hair removal trauma. Physical exam revealed erythematous papules, either intact or with central umbilication, that evolved into crater-like ulcerations surrounded by an erythematous halo and with a dark, central keratotic plug. Intense xerosis of the skin was associated (Figure 1, Figure 2). Histopathology showed cup-shaped epidermal ulcerations filled with crusts and keratotic material, and the transepidermal elimination of collagen and elastic fibers (Fig. 3).

**Figure 1:**
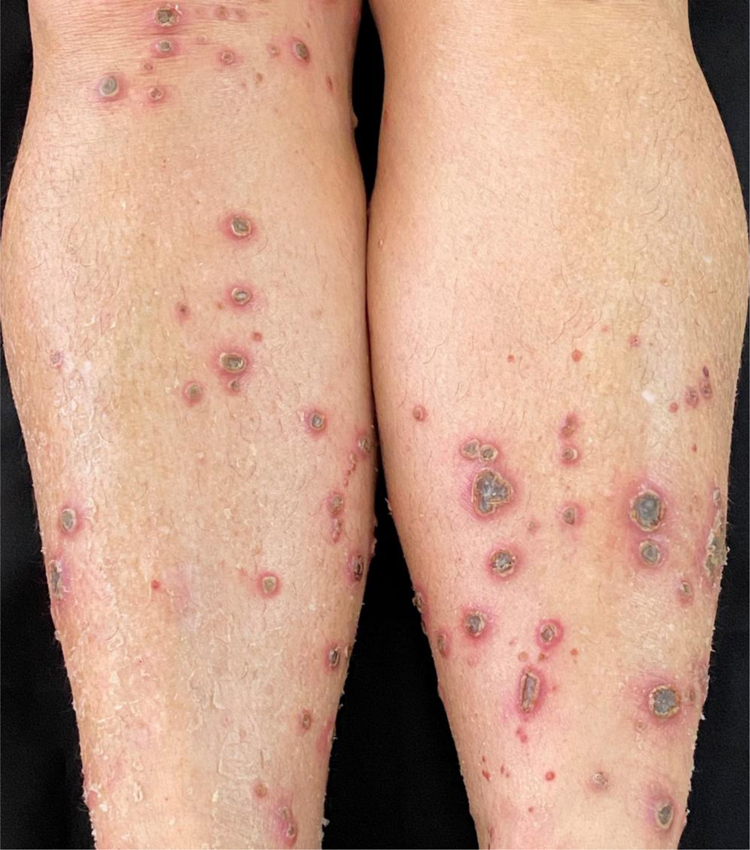
Multiple erythematous papules and crater-like ulcerations on lower limbs with associated xerosis.

**Figure 2:**
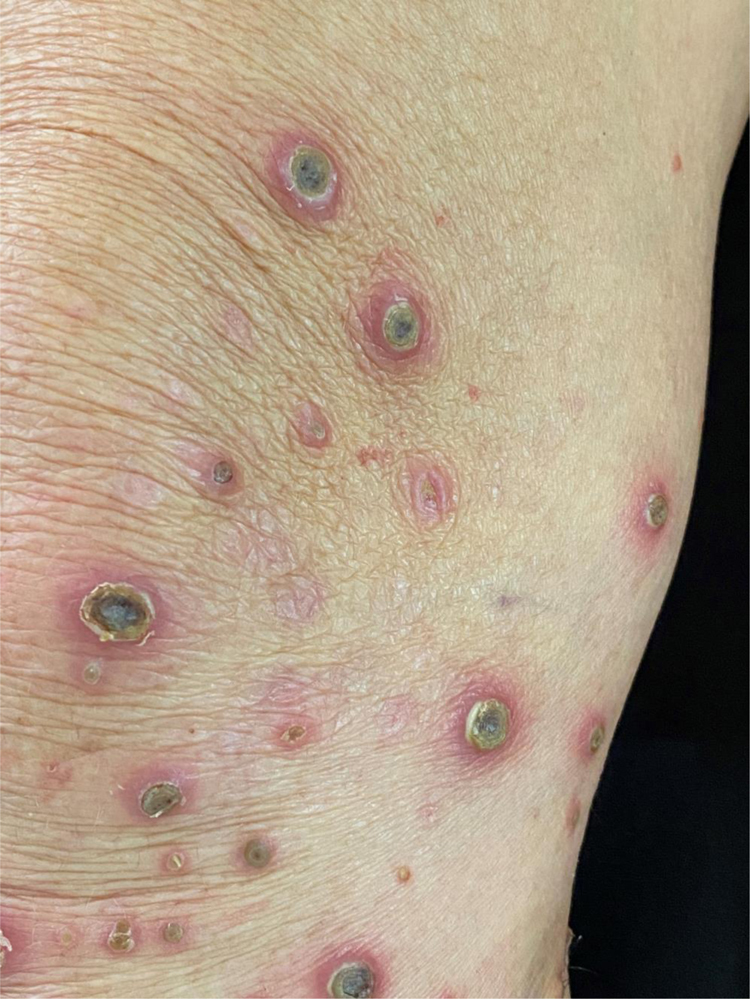
Crater-like ulcerations with a central keratotic plug.

**Figure 3:**
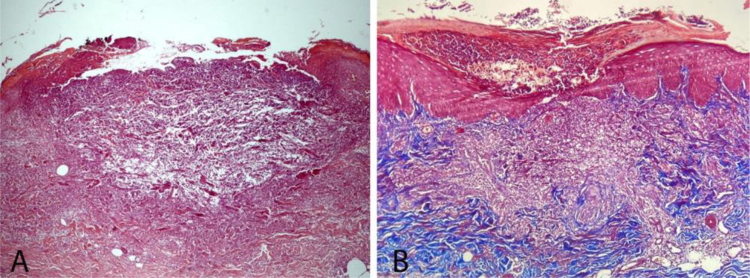
(A), Histopathological exam showing a cup-shaped lesion containing keratin, cellular debris, neutrophils and collagen and elastic fibers (Hematoxylin & eosin, ×50). (B), Presence of intraepidermal collagen fibers perpendicular to the skin surface. (Masson’s trichome, ×40).

Perforating dermatoses are a rare and heterogenous group of diseases characterized by transepidermal elimination of dermal compounds, mainly collagen and elastic fibers. The four classical or primary forms are represented by Kyrle’s disease, elastosis perforans serpiginosa, perforating folliculitis, and reactive perforating collagenosis. The word “primary” implies that the transepidermal elimination is the main pathological process. On the other hand, in perforating dermatoses termed “secondary” (granuloma annulare, necrobiosis lipoidica, rheumathoid nodule, lichen nitidus, chondrodermatitis nodularis helicis, etc.), perforation of the epidermis is regarded as an epiphenomenon.^1^ With the exception of elastosis perforans serpiginosa and the extremely rare familial reactive perforating collagenosis (OMIM nº 216700), that may start in childhood or adolescence, the primary forms are classically diseases of adulthood.

In 1989, Rapini et al. proposed the umbrella term “Acquired Perforating Dermatosis” (APD) to encompass cases with onset in adult life and associated with diabetes mellitus and chronic renal failure, especially in patients on dialysis.^2^ Later on, it became evident that other comorbidities such as cardiac, rheumatological, pulmonary, and malignant disorders, among others, can also be associated with APD.^3^

Based on clinical and histopathological features, the diagnosis of Acquired Reactive Perforating Collagenosis (ARPC), triggered by excoriation of pruriginous lesions, was made. In the two largest case series published thus far, ARPC was the most prevalent type of APD.^3^, ^4^ The clinical presentation of erythematous or brownish papules, plaques or nodules that soon become umbilicated and evolve into a crater-like ulceration with a central keratotic plug is characteristic of this condition. Lesions favor the extensor surfaces of the limbs, gluteal area, and trunk. Mucosal involvement is only observed in familial reactive perforating collagenosis.^3^, ^4^

The etiopathogenesis of all APD variants remains uncertain. The ample spectrum of associated disorders (diabetes mellitus and other endocrinological diseases, chronic renal failure, malignancies, medications, etc.) renders it difficult to develop a unicist concept to explain the etiopathogenesis. It is speculated that the impairment of metabolic products elimination in chronic renal failure and the microangiopathy in diabetes mellitus may play a role. The relationship of pruritus, excoriation and the onset of the lesions is supported by the common finding of linear lesions (Köebner phenomenon), as well as the exclusive location of the lesions within ones hand reach.^1^, ^5^

Our patient, with no comorbidity or medication usage, was diagnosed as ARPC triggered both by the excoriation of the pruriginous lesions and the xerosis of the lower limbs.

## Financial support

None declared.

## Authors' contributions

João Renato Vianna Gontijo: Conception and design of the study; acquisition of data; drafting the article or revising it critically for important intellectual content; final approval of the version to be submitted.

Florentino Fernandes Júnior: Drafting the article and revising it critically for important intellectual content; final approval of the version to be submitted.

Luciana Baptista Pereira: Drafting the article and revising it critically for important intellectual content; final approval of the version to be submitted.

Moisés Salgado Pedrosa: Drafting the article and revising it critically for important intellectual content; final approval of the version to be submitted.

## Conflicts of interest

None declared.
